# Artificial Intelligence in Public Health Education: A Scoping Review of Workforce Competency Development

**DOI:** 10.1002/hsr2.72066

**Published:** 2026-03-09

**Authors:** Mst Masuma Akter Semi, Srabani Das, Mashuk Rahman Utsho, Adib Hossain, Md Bayzid Kamal, Arif Ahmed Sizan, Afia Fairooz Tasnim, Sumaiya Yeasmin, Mst. Rina Parvin

**Affiliations:** ^1^ Department of Education Westcliff University California USA; ^2^ Department of Business Administration St. Francis College New York USA; ^3^ Department of Business Analytics Trine University Indiana USA; ^4^ Department of Business Analytics Brooklyn College New York USA; ^5^ Department of Business Administration Westcliff University California USA; ^6^ Department of Public Health California State University Long Beach California USA; ^7^ Department of Psychology St. Francis College New York USA; ^8^ Combined Military Hospital Dhaka Bangladesh

**Keywords:** artificial intelligence, digital literacy, public health education, workforce competency

## Abstract

**Background:**

Artificial intelligence (AI) has catalyzed profound shifts in public health education, compelling institutions to explore innovative methods to prepare a digitally competent and resilient workforce. AI has emerged as a transformative tool, enabling adaptive, personalized, and scalable learning experiences. However, the long‐term implications and equity considerations of AI integration in education remain underexplored.

**Aim:**

This scoping review aimed to map the existing literature on the role of AI in public health education, focusing on its impact on workforce competency development and associated challenges.

**Methods:**

Following Arksey and O'Malley's framework with enhancements from Levac et al., a comprehensive literature search was conducted across major databases, including PubMed, Scopus, and IEEE Xplore. Eligible studies, published from January 2015 to May 2025, were screened using PRISMA‐ScR guidelines. Data were extracted and thematically analyzed to identify patterns, competencies addressed, and ethical or institutional considerations.

**Results:**

A total of 26 studies were included. Key themes included the transformation of pedagogical practices through AI‐powered simulations and adaptive platforms, the rise of AI‐specific and digital literacy training, institutional disparities in readiness, and significant ethical concerns around algorithmic bias and equitable access. Interdisciplinary collaboration and curriculum reform were identified as pivotal in sustaining AI integration.

**Conclusion:**

AI holds great promise in enhancing public health education, but its integration should be approached with attention to equity, institutional capacity, and ethical responsibility. Strategic policy, curriculum reform, and ongoing research are critical to fostering a workforce equipped for future public health challenges.

## 1 Introduction

The landscape of education has undergone dramatic transformation, particularly within the field of public health, where urgent adaptation was needed to address evolving workforce demands [1]. Traditional methods of teaching were rapidly re‐evaluated as institutions sought innovative strategies to deliver effective education under unprecedented constraints [2]. Amid this transformation, artificial intelligence (AI) emerged not merely as a technological trend but as a foundational pillar in reimagining how public health professionals are trained and supported [3]. From virtual simulations and intelligent tutoring systems to adaptive learning platforms and data‐driven decision support tools, AI has provided flexible, scalable, and personalized learning opportunities that were otherwise impossible in conventional classroom settings [4]. These tools have not only helped to maintain continuity in education during disruptions but have also opened doors for more efficient and dynamic competency development across diverse learner populations [5].

As AI technologies continue to advance, their integration into public health education has extended beyond short‐term solutions into long‐term pedagogical strategies [6]. Institutions are increasingly recognizing the potential of AI to enhance digital literacy, foster analytical skills, and embed ethical reasoning into curricula tailored for the next generation of public health professionals [7]. Educational frameworks have started to shift toward interdisciplinary models, combining data science, behavioral insights, and technological fluency to meet the growing complexity of real‐world public health challenges [8]. This shift reflects a deeper understanding that workforce competency is not limited to knowledge acquisition but also includes the ability to navigate digital ecosystems, interpret machine‐generated insights, and respond to ethically nuanced scenarios [9]. As such, AI's role has transitioned from being a supplementary asset to a core enabler of resilient, future‐oriented public health education [10].

Nevertheless, the integration of AI in public health education is not without its challenges. Concerns about equity, access, institutional readiness, and ethical implications underscore the need for intentional and inclusive design in AI‐enhanced learning environments [11]. The rapid pace of technological advancement has highlighted disparities in digital infrastructure and faculty preparedness, particularly in low‐resource settings where educational innovation may be hampered by logistical and policy‐related limitations [12]. Simultaneously, ethical questions surrounding algorithmic bias, data security, and informed consent remain unresolved, raising concerns about the unintended consequences of AI adoption [13]. These challenges emphasize the necessity for robust governance frameworks and collaborative strategies that prioritize transparency, accountability, and inclusiveness in AI implementation [14, 15]. Addressing these concerns is critical to ensuring that technological advancement does not inadvertently widen existing gaps in education or public health capacity [16, 17].

In this context, it becomes imperative to map and synthesize how AI has been utilized within public health education to support workforce competency development. Understanding the scope, trends, and limitations of current literature in this area will provide valuable insights for educators, policymakers, and researchers aiming to align technological innovation with workforce needs. This scoping review was therefore conducted to explore the breadth of existing evidence on the integration of AI in public health education, identify emerging themes and challenges, and assess its contributions to developing a digitally competent, ethically aware, and practice‐ready public health workforce.

## 2 Methodology

### 2.1 Study Design

This scoping review was conducted to explore how artificial intelligence (AI) is utilized in public health education to support the development of workforce competencies. The methodological approach followed the frameworks proposed by Arksey and O'Malley (2005), with enhancements introduced by Levac and colleagues [18]. This design was selected because it is particularly well‐suited for mapping complex and emerging areas of research, such as the intersection of AI and public health education. The focus of this review was to examine the breadth and nature of existing literature, identify key themes, and uncover knowledge gaps rather than evaluating intervention effectiveness, which distinguishes scoping reviews from systematic reviews.

Given the fast‐evolving and interdisciplinary nature of AI in educational contexts, the scoping review allowed for the inclusion of a diverse range of evidence types, encompassing empirical research, conceptual discussions, and gray literature. This comprehensive scope helped build a holistic understanding of how AI is influencing educational practices and competency frameworks in public health. The review was structured around the following research question: “How has artificial intelligence been utilized in public health education to enhance workforce competency?” This question guided all stages of the review, including study selection, data extraction, and synthesis.

### 2.2 Eligibility Criteria

A clear set of inclusion and exclusion criteria was established to ensure that only relevant and meaningful literature was included in the review. Studies were eligible if they focused on individuals involved in public health education or training, such as students, instructors, or professionals, and if they addressed the integration or application of AI technologies within educational settings. Additionally, included studies needed to highlight outcomes related to workforce competency development, which could include skills such as critical thinking, digital literacy, clinical reasoning, or data analysis. The context of the studies was also a determining factor. Only those published from 2015 onward were considered to capture the most recent shifts in educational strategies. Furthermore, studies had to be published in English and could include a wide range of publication types, including peer‐reviewed articles, conference papers, institutional reports, and high‐quality white papers.

### 2.3 Information Sources and Search Strategy

To capture a wide array of literature, a comprehensive and systematic search strategy was devised in collaboration with an experienced research librarian. Several electronic databases were utilized, including PubMed, Scopus, Web of Science, CINAHL, ERIC, and IEEE Xplore, with Google Scholar employed to locate relevant gray literature (Table 1). The search covered literature published between January 2015 and May 2025, reflecting the timeframe of interest for recent developments in the field. Boolean operators and truncation symbols were used to refine searches and maximize the relevance of retrieved studies. Core search terms included various combinations and synonyms of “artificial intelligence,” “machine learning,” “public health education,” “competency,” and “training.” For example, one of the search strings used in PubMed was: (“artificial intelligence” OR “machine learning” OR “AI”) AND (“public health education” OR “health training” OR “curriculum”) AND (“competency” OR “skills” OR “capacity”). In addition to database searches, the reference lists of selected articles were manually screened to uncover studies not identified in the initial search. Gray literature and policy documents were also considered to enrich the review with practical insights and contextual information often absent in academic publications.

**TABLE 1:** Search strategy and sources.

Database	Search string	Filters applied	Notes
PubMed	(“artificial intelligence” OR “machine learning” OR “AI”) AND (“public health education” OR “health training” OR “curriculum”) AND (“competency” OR “skills” OR “capacity”)	English; Human; Peer‐reviewed	Boolean operators and truncation symbols used; Manual reference check
Scopus	(“artificial intelligence” OR “machine learning” OR “AI”) AND (“public health education” OR “curriculum”) AND (“competency” OR “skills”)	English; Article type: Journal and Conference Paper	Search strategy adapted for Scopus syntax
Web of Science	TS = (“artificial intelligence” OR “machine learning”) AND TS = (“public health education”) AND TS = (“competency”)	English; Document Type: Article	Topic search used; Refined by research areas
CINAHL	(“artificial intelligence” OR “AI”) AND (“public health education” OR “nursing education”) AND (“digital literacy” OR “competency”)	English; Academic Journal; Human	Used CINAHL Subject Headings
ERIC	(“artificial intelligence” OR “machine learning”) AND (“health education”) AND (“skills” OR “training”)	Peer‐reviewed only; English	Search refined using ERIC thesaurus
IEEE Xplore	(“artificial intelligence” OR “machine learning”) AND (“public health education” OR “training”) AND (“skills” OR “competency”)	Journals, Conferences; English	Engineering/technical filters applied
Google Scholar	“artificial intelligence” “public health education” “competency”	English; Title screening for relevance	Used for gray literature and policy documents

### 2.4 Study Selection

**FIGURE 1:**
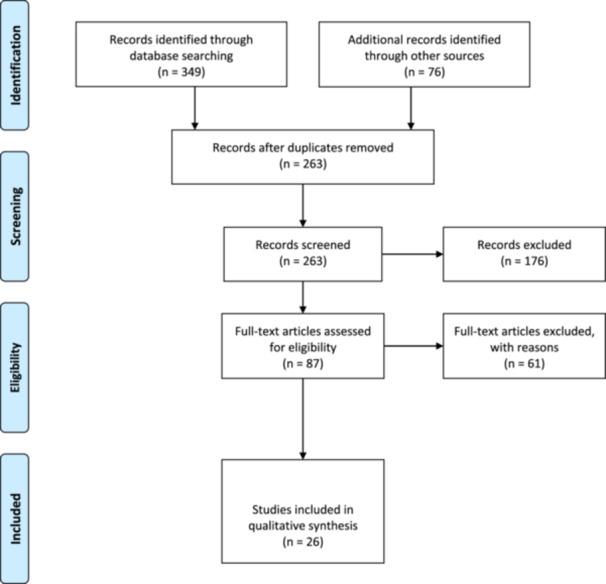
PRISMA flowchart.

All identified records were imported into EndNote 20 for reference management, and duplicate entries were systematically removed. The selection of studies was carried out in two stages to ensure methodological rigor. Initially, two reviewers independently screened the titles and abstracts of retrieved studies against the predefined inclusion criteria. Discrepancies between reviewers were resolved through discussion or, if necessary, consultation with a third reviewer to reach a consensus. Following the initial screening, full texts of studies deemed potentially eligible were retrieved and thoroughly reviewed. At this stage, further exclusions were made if studies did not meet the criteria upon closer inspection. Reasons for exclusion were documented to maintain transparency. These included studies that lacked a clear focus on public health education, did not involve the use of AI technologies, failed to address workforce competency, or were editorials without empirical or conceptual contributions. The entire study selection process was recorded using the PRISMA‐ScR (Preferred Reporting Items for Systematic Reviews and Meta‐Analyses extension for Scoping Reviews) flowchart (Figure 1) to illustrate the number of records identified, screened, included, and excluded throughout the process [19].

### 2.5 Data Charting and Extraction

A standardized data charting form was developed and pilot‐tested using five randomly selected studies to ensure its clarity and comprehensiveness. Once finalized, the form was used to systematically extract relevant data from each included study. The extracted information included bibliographic details (author, year, and country), study design, target populations, the type of AI technology used (such as natural language processing, machine learning, or virtual simulation), and the educational context in which the technology was applied. Other key data points included the specific competency domains addressed, such as epidemiological modeling, analytical reasoning, or digital health skills, as well as findings related to workforce development outcomes. Any challenges, limitations, or ethical considerations discussed by the authors were also noted, along with recommendations for future practice and research (Table 2). Data extraction was performed independently by two reviewers, with discrepancies resolved through discussion or by involving a third reviewer. This dual‐extraction approach minimized the risk of bias and enhanced the reliability of the synthesized data.

**TABLE 2:** Summary of included studies.

Author (year)	Country	Study design	Target population	AI technology used	Educational context	Competency domains	Workforce development outcomes	Challenges/limitations/ethical considerations	Recommendations for public health education
Jungwirth & Haluza, 2023	Austria	Exploratory study	General public and public health stakeholders	GPT‐3 (Natural Language Processing, Machine Learning)	Public health research and education	Evidence‐based reporting, digital communication, public health analysis	Enhanced public health reporting, automation of summaries, support for health education materials	Invented references, lack of traceability, consent/authorship concerns, ethical implications of AI contributions	Use AI as support tools, promote transparency, update policies, ensure ethical usage
Katirai et al., 2023	Japan	Exploratory qualitative workshop	Patient and Public Involvement Panel (11 participants)	General AI in Healthcare (Machine Learning, NLP, Robotics, etc.)	Public education and stakeholder engagement through participatory workshops	Ethical understanding, healthcare system literacy, digital engagement	Improved patient and public understanding; potential for enhanced engagement	Concerns about loss of autonomy, data management, technical reliability, equity, healthcare costs	Include patient/public voices in AI development; improve AI literacy; foster balanced dialog around expectations and concerns
Deiana et al., 2023	Italy	Qualitative and quantitative evaluation study	General public (via simulated ChatGPT responses to WHO myths)	ChatGPT (GPT‐3.5 and GPT‐4.0)	Public Health Information, Health Literacy	Information Literacy, Critical Thinking	Improved health communication analysis, potential AI integration in public health	Risk of misinformation, digital divide, unequal access to AI tools, lack of source transparency	Integrate AI tools with expert oversight, address digital inequality, promote critical appraisal skills
Seth et al., 2023	Canada, United States	Viewpoint	Undergraduate medical students	Machine learning, neural networks, clinical decision support systems, large language models	Undergraduate Medical Education (UME)	Data science fundamentals, health data sources, data analysis, ethics and cybersecurity, clinical application of AI tools	Enhanced data literacy, ability to use and evaluate AI tools in clinical care, readiness for data‐driven decision‐making	Faculty knowledge gaps, curricular integration, ethical concerns (privacy, fairness, equity), evolving content, ideological barriers	Introduce data science early, Longitudinal integration across curriculum, Faculty development, Emphasize equity and real‐world application
Abou Hashish & Alnajjar, 2024	Saudi Arabia	Descriptive cross‐sectional correlational	266 undergraduate nursing students (third & fourth year)	General AI applications (clinical decision support, digital transformation tools)	Undergraduate nursing education	Digital knowledge, digital skills, digital health literacy, attitudes toward AI	Positive correlations among digital knowledge, skills, DHL, and AI attitudes; seniors showed higher competency	Inadequate user skills; limited budget & resources; information security & privacy concerns	Integrate hands‐on digital health & AI training into curricula; provide targeted internet‐use skill courses
MacIntyre et al., 2023	Australia, United Kingdom	Narrative review	Public health systems and authorities	Natural Language Processing (NLP), Machine Learning, BERT, Geospatial Forecasting	Public health epidemic surveillance and early warning systems	Epidemiological modeling, outbreak detection, data curation, risk analysis	Enhanced early detection of epidemics, improved decision‐making for outbreak response	Data censorship, human moderation bias, limited public access to some systems	Widespread adoption of AI‐based surveillance, training on digital surveillance tools, integrate AI‐based risk analysis into education
Mun et al., 2024	Australia	Program redesign/viewpoint (qualitative descriptive)	Postgraduate health professionals and students interested in digital health transformation	Digital health technologies, including machine learning, artificial intelligence, electronic health records, virtual care	Graduate Certificate program in Digital Transformation of Health, University of Melbourne	AMIA clinical informatics domains; Australian Health Informatics Competency Framework domains	Developing digitally capable health workforce; leadership and change management skills	Rapidly evolving field requires continuous update; diverse learner backgrounds; need co‐design with stakeholders; maintaining curriculum currency	Align programs with competency frameworks; include advanced AI & data science content; provide flexible electives; integrate interdisciplinary learning and leadership training
Russell et al., 2023	USA	Qualitative study (expert interviews and thematic analysis)	Health care professionals (including physicians, nurses, and pharmacists) and AI/ethics experts	AI‐based tools (machine learning, deep learning, NLP, neural networks)	Health professions education; clinical environments; competency‐based training	Basic knowledge of AI, Social and ethical implications, AI‐enhanced clinical encounters, Evidence‐based evaluation, Workflow analysis, Practice‐based learning	Defined 6 AI competency domains and 25 subcompetencies for ethical and effective AI tool use in clinical settings	Potential bias, data representativeness, equity concerns, ethical responsibility, workflow disruption, rapid AI advancement	Develop competency‐based curricula, include ethics, promote interdisciplinary collaboration, continuous learning, engage diverse perspectives
Lomis et al., 2021	United States	Discussion paper/expert perspective	Health professions educators and learners	Various AI tools (machine learning, conversational agents, robotics, predictive analytics)	Health professions education programs (medicine, nursing, allied health)	AI literacy, data stewardship, communication, ethics, systems thinking	Preparedness to leverage AI, improved teaching efficiency, interprofessional collaboration	Curriculum overload; lack of faculty expertise; bias in data/algorithms; resource constraints	Integrate hands‐on digital health & AI training into curricula; foster interprofessional learning; develop faculty capacity; leverage AI for adaptive learning and administration.
Ng et al., 2023	Hong Kong	Conceptual paper/framework development	University teachers/educators	AI‐driven learning platforms, intelligent tutoring systems, chatbots, facial recognition, predictive analytics, adaptive systems	Higher Education/Post‐pandemic Online Teaching	Technological knowledge, pedagogical knowledge, ethical awareness, content knowledge, professional engagement	Improved teaching efficiency, personalized learning support, administrative task automation, enhanced student engagement and self‐regulation	Lack of technical readiness, ethical concerns (e.g., surveillance, privacy), AI misunderstandings/misleading recommendations, fear of teacher replacement	Provide teacher training programs; upgrade digital infrastructure; incorporate AI and digital skills in curricula; develop holistic digital competency frameworks, including life, career, and ethical skills
Alenezi et al., 2024	Saudi Arabia	Quantitative study using Partial Least Squares Structural Equation Modeling	329 nurses from five hospitals in Riyadh Province	AI‐assisted diagnostics, decision support systems	Hospital‐based clinical settings	Nursing workforce competencies (knowledge, attitude, skills in AI and technology use)	Technology integration positively influenced productivity; AI initially disrupted but later supported productivity; competencies mediated outcomes	Initial productivity drop due to AI disruption; leadership had no significant moderating effect; self‐reported data; limited to Riyadh	Develop comprehensive training programs on digital tools; foster a culture of innovation; regularly evaluate technology impact; provide technical support
Nasseef et al., 2022	Saudi Arabia	Quantitative – survey‐based (SEM analysis)	Healthcare CEOs, senior managers, doctors, nurses, and healthcare practitioners involved in COVID‐19 decision‐making	Machine learning, computer‐aided diagnosis, deep learning, natural language processing, chatbots	Public healthcare system (Ministry of Health), training via apps (e.g., Seha, Tetamman, Tabaud)	Decision‐making, diagnosis, problem‐solving, experience‐based reasoning	Improved decision quality, efficiency, accuracy, better mental representation for problem‐solving	Need for more resources, data accessibility issues, lack of pre‐existing familiarity with AI tools	Enhance experience‐based decision‐making, develop regulatory frameworks, expand AI training and awareness
Sharifi et al., 2021	Iran, China, USA, Germany, Malaysia, Qatar, and so forth	Narrative review	Industries, health sector, education sector, energy consumers	Machine Learning, Intelligent Sensors, Mobile Technology, IoT, Cloud Computing, Virtual Reality, Deep Learning, AI‐based Surveillance, Blockchain	Distance Education, Remote Health Monitoring, Virtual Training for Medical Staff, Mobile Health Applications	Digital Health Skills, Remote Monitoring, Epidemiological Modeling, Data Analysis, AI Literacy	Preparedness for Future Pandemics, Enhanced Energy Management, Improved Medical Diagnosis, Enhanced Agricultural Efficiency	Technical limitations, ICT infrastructure issues, Socio‐economic inequality, Security risks, Privacy concerns, High cost, Lack of awareness	Promote ICT use in education, Integrate AI in health curriculum, Encourage government digital education programs, Develop monitoring systems, Increase public awareness
Haneef et al., 2021	France	Case study using Supervised Machine Learning	Adults aged 18–69 from CONSTANCES cohort (*n* = 44,659)	Supervised Machine Learning (Linear Discriminant Analysis)	Public health surveillance, epidemiological cohort linkage	Data analytics, health informatics, algorithm development	Algorithm for diabetes incidence estimation, moderate accuracy	Limited elderly population, low specificity, unbalanced data, lack of clinical input, computational demands	Enhance AI capacity in public institutions, integrate clinician expertise, explore variable time windows, apply ML for risk factor analysis
Tornimbene et al., 2025	Multi‐country (Germany, USA, Australia, Canada, France, Senegal)	Meeting report/forum proceedings	Public health professionals, policymakers, researchers	Machine Learning, NLP, Predictive Modeling, Large Language Models, Image Processing	Public health forums, interdisciplinary training, healthcare systems	Data analysis, outbreak management, diagnostics, epidemic surveillance	Improved epidemic preparedness, enhanced diagnostic capabilities, informed public health decision‐making	Data privacy, algorithmic bias, lack of diverse data, human‐AI interaction, ethical governance	Promote inclusive AI design, develop ethical frameworks, integrate multidisciplinary collaboration, enhance equity and governance
Rojahn et al., 2023	United States	Cross‐sectional survey study	General American public (*N* = 203)	General medical AI (e.g., diagnostic algorithms, virtual nurse agents)	Public education and awareness in healthcare settings	Trust, decision‐making, cultural bias understanding, privacy concerns	Not directly assessed; insights into public acceptance for future integration	Distrust, algorithm aversion, perceived cultural bias, data privacy, lack of understanding of AI (“black‐box”)	Enhance public trust through transparency, education on AI functionality, involve human oversight, integrate AI as a support tool
Asal et al., 2025	Egypt	Cross‐sectional survey	Nurse educators (*n* = 600) across Egyptian nursing faculties	General AI‐driven tools discussed (virtual simulations, adaptive learning platforms); measured via AI Readiness Scale	Undergraduate and graduate nursing programs in university faculties	Digital competence (technical, pedagogical, ethical) & AI readiness (cognitive, ability, vision, ethics)	Higher digital competence and AI readiness predicted greater pedagogical innovation, implying better preparedness for technology‐integrated teaching	Limited formal AI training, infrastructure constraints, variable institutional support; ethical need for privacy and responsible AI use; cross‐sectional design limits causality	Integrate hands‐on digital health and AI training into curricula; foster interprofessional learning; develop faculty capacity; leverage AI for adaptive learning and administration
Yan et al., 2025	China	Observational quantitative study with predictive modeling and clustering	Vocational education students (*n* = 1200)	AI‐driven training, Machine Learning (Random Forest, Linear Regression), KMeans clustering	Higher vocational education	Competency assessment, student engagement, demographic analysis	Improved competency scores, identification of learner profiles, gender and training level effects	Lack of qualitative insights; potential algorithmic bias; omission of socioeconomic variables; limited generalizability	Integrate personalized AI training; ensure data fairness and diversity; apply clustering for adaptive learning; expand AI to broader educational contexts
Panteli et al., 2025	Multinational (EU, WHO Europe, UK, Germany, Portugal, Malta)	Viewpoint/policy perspective	Public health institutions and policymakers	Machine Learning, Natural Language Processing, Clustering, Chatbots, XAI	Public Health Workforce Training and Institutional Readiness	Digital Literacy, Data Governance, AI Literacy, Cybersecurity, Ethical AI Use	Increased readiness, skills development, intersectoral collaboration, AI capacity‐building	Bias, equity, data privacy, lack of infrastructure, cybersecurity risks, workforce skill gaps	Develop robust legal/ethical AI frameworks, invest in secure digital infrastructure, train public health professionals, prioritize equity, promote explainable AI, ensure stakeholder engagement, address environmental impact of AI
Salem et al., 2025	Saudi Arabia, Egypt, Jordan, India, Pakistan, Philippines	Cross‐sectional study	Medical educators in higher education	General AI tools for education (ADELE framework)	Medical education in developing nations	AI Awareness, Development of AI Skills, AI Efficacy, Leanings Toward AI, AI Enforcement	Improved AI readiness and competency among educators; emphasis on ethical AI use	Lack of AI‐trained faculty, limited infrastructure, cultural influences on ethical perceptions, insufficient hands‐on training	Integrate AI into medical curricula, provide hands‐on training, address ethical implications, use the ADELE framework for competency development
Sun, 2021	China	Qualitative multi‐case study	Doctors, patients, hospital managers, IT firm staff	Receptionist robot, voice‐based EMR, diagnostic AI, medical imaging AI	Professional healthcare settings (3 hospitals)	Understanding AI systems, diagnostic skills, data annotation, EMR use	Improved efficiency, reduced misdiagnosis, enhanced diagnostic skills, exposure to AI	Low AI adoption, increased workload, limited AI‐hospital system integration, data quality/quantity limitations in community hospitals	Leverage expert and legitimate power structures, integrate AI training for staff, use informal knowledge‐sharing strategies, adapt strategies to AI complexity
Jiao et al., 2023	China	Review	Public health systems and populations during epidemics	AI, Big Data, NLP, Machine Learning, Deep Learning, Imaging AI, Robotics	Not directly educational, but focuses on public health training, policy guidance, and AI integration	Epidemic surveillance, data analytics, telemedicine, AI policy, resource allocation, diagnosis support	Improved response capabilities, enhanced telehealth access, more efficient diagnosis and treatment, better policy planning	Data quality, privacy, lack of unified data schema, model limitations, rural data gaps, policy effectiveness estimation	Develop AI‐integrated prevention systems; legislate data use; promote multidisciplinary collaboration; enhance data sharing platforms; foster international cooperation
Witkowski et al., 2024	USA (Florida)	Exploratory mixed‐methods	600 adults from Florida, USA	AI in patient administration, clinical decision support, patient monitoring, healthcare interventions	Public health and medical ethics education context, survey conducted for understanding public perception	Decision Self‐Efficacy, Patient‐centered care, Digital Literacy	Enhanced understanding of patient confidence and comfort with AI; implications for health workforce training	Lack of human touch, distrust in AI, gender and age differences in acceptance, concerns over cost, bias, and transparency	Include AI‐related informed consent; use AI as supportive tool; educate healthcare providers on ethical AI implementation; begin AI use in non‐relational tasks
Chiappelli et al., 2018	USA, Israel	Hypothesis/conceptual framework	General population (focus on viral infections like SFTS)	AI‐aided immune tweening, biostatistical regression modeling	Translational health care, public health immunization strategies	Data analysis, Immunological modeling, AI in public health	Enhanced understanding and predictive modeling of immune response; improved immunization strategies	Knowledge gaps in immune response variables; need for AI model validation; emerging viral threats	Incorporate AI and bioinformatics in immunization program planning; foster interdisciplinary modeling approaches
Gray et al., 2022	Australia	Expert survey (mixed methods: quantitative + qualitative thematic analysis)	Health workforce educators and training professionals (*N* = 39)	Machine learning, NLP, robotics, rule‐based expert systems, RPA	Formal education, CPD, specialist training, organizational strategy	Ethics, machine learning, data suitability, human‐machine interaction, diagnosis/treatment apps	Strategic planning, curriculum reviews, CPD programs, training initiatives	Lack of governance, resource constraints, cultural unreadiness	Integrate hands‐on AI training, interprofessional education, develop governance, tailor training to roles
Wu et al., 2023	Multiple (Netherlands, India, Canada, USA, China, Belgium, Germany)	Meta‐synthesis of qualitative studies	General public and patients in various healthcare settings	Various AI applications, including imaging, mHealth apps, robotic surgery, telepsychiatry	Not directly educational, but includes perceptions of AI literacy and data transparency	Health literacy, digital competency, ethical awareness	Insights into patient engagement and AI‐assisted decision‐making	Privacy concerns, accountability, data bias, overreliance on AI, loss of human connection	Enhance AI transparency, train healthcare workers to use AI as supportive tool, public education on AI benefits and limitations

### 2.6 Data Synthesis and Analysis

**FIGURE 2:**
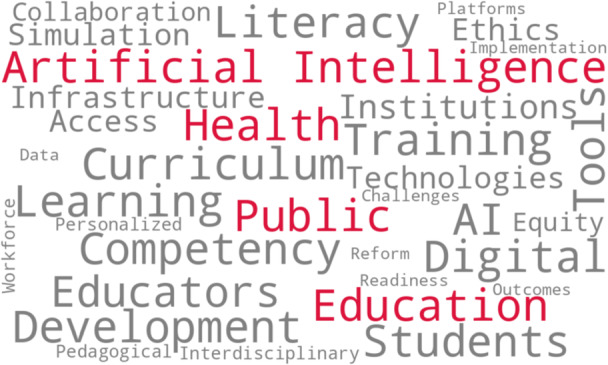
Thematic word cloud depicting core concepts in AI‐enhanced public health education.

**FIGURE 3:**
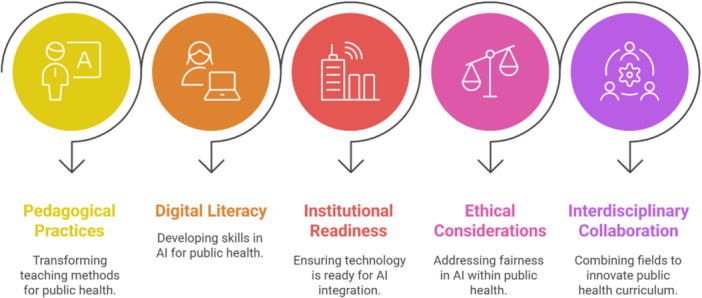
AI integration in public health education.

After data extraction, a descriptive and thematic synthesis was conducted to integrate and interpret the findings. Quantitative synthesis included basic counts and categorizations, such as the frequency of AI applications, study locations, or participant groups. Qualitative data were analyzed using Braun and Clarke's (2006) framework for thematic analysis [20], which involves a systematic process of familiarization with the data, generating initial codes (Figure 2), identifying recurring themes, reviewing and refining themes (Figure 3), and ultimately defining them in relation to the research question. The goal of the thematic analysis was to uncover meaningful patterns and insights regarding how AI has contributed to workforce competency development in public health education. Themes were developed collaboratively among the review team to ensure consistency and validity. This process allowed the review to identify both the opportunities and limitations associated with integrating AI in educational strategies aimed at preparing a competent and future‐ready public health workforce.

## 3 Results

### 3.1 Summary of Included Studies

This review synthesizes findings from 26 studies [21, 22, 23, 24, 25, 26, 27, 28, 29, 30, 31, 32, 33, 34, 35, 36, 37, 38, 39, 40, 41, 42, 43, 44, 45, 46] across diverse global contexts, exploring how artificial intelligence (AI) is transforming public health education and workforce development. Various AI technologies, such as natural language processing, machine learning, neural networks, and robotics, have been deployed across diverse regions, including North America, Europe, Asia, and the Middle East. These technologies facilitated enhancements in public health reporting, communication, personalized education, and clinical decision‐making. Many studies highlighted improvements in teaching efficiency, student engagement, and healthcare professionals' preparedness for data‐driven decision‐making. Notably, AI integration also contributed to epidemic preparedness, mental health management, and immunization strategies. Furthermore, readiness for digital transformation and competency in AI applications were significantly enhanced through targeted training and experiential learning. Of the 26 studies reviewed, 9 were qualitative or exploratory studies (e.g., interviews, workshops, and expert perspectives), 6 employed quantitative methods (e.g., surveys, PLS‐SEM, and predictive modeling), 5 used mixed‐methods or expert surveys, 3 were conceptual or framework development papers, 2 were narrative or descriptive reviews, and 1 was a program redesign study. No experimental or longitudinal studies were included.

To effectively embed AI into public health education, the studies recommend a range of strategic actions. These include developing competency‐based and ethically grounded curricula, fostering interdisciplinary collaboration, and incorporating hands‐on digital health training. Several studies emphasized the need for early and longitudinal integration of data science and AI into curricula, strengthening faculty capacity, and addressing digital inequality. Others advocated for inclusive design, improved AI literacy, and public engagement to foster trust and equity. Moreover, leveraging explainable AI, secure digital infrastructure, and policy frameworks were suggested to support sustainable implementation. Collectively, these recommendations underscore the importance of aligning educational programs with evolving technological needs while upholding ethical standards and promoting inclusive, transparent, and adaptive learning environments.

### 3.2 Transformation of Pedagogical Practices in Public Health Education

The integration of artificial intelligence (AI) has reportedly influenced the evolution of teaching methods within public health education, as observed across the included studies. Educational institutions have increasingly embraced AI‐assisted learning environments, such as virtual simulations, intelligent tutoring systems, and adaptive learning platforms [30]. These tools facilitated personalized learning by dynamically adjusting content and pace according to individual student performance, learning preferences, and real‐time feedback [22]. AI‐powered virtual simulations played a pivotal role in replicating real‐world public health scenarios [27]. These immersive tools provided learners with opportunities to engage in epidemiological modeling, public health intervention planning, and policy decision‐making in virtual environments [35]. The simulations enabled students to develop critical thinking and problem‐solving skills without being constrained by physical limitations [43].

Moreover, AI‐integrated platforms, including automated discussion facilitators and real‐time feedback systems, were widely adopted to support educators and reduce workload [24, 37]. These tools enhanced student engagement in blended learning formats [34, 42]. Learner performance outcomes were reported to show improvement when interactive AI technologies were embedded into pedagogical strategies [36]. However, implementation challenges persisted, particularly related to faculty resistance, insufficient training on AI tools, and disparities in technological access across regions, especially in lower‐resource settings [23, 45]. While these observations stem from descriptive and exploratory studies included in this review, more rigorous pedagogical research will be needed to establish causal links between AI use and teaching effectiveness.

### 3.3 Digital Literacy and AI‐Specific Competency Development

The emergence of AI in public health education underscored the urgent need to enhance digital literacy and build AI‐specific competencies among both educators and learners. Recent years have revealed significant variations in digital preparedness, with technological skills becoming essential for future public health professionals [37, 46]. Curricular reforms began to emphasize core competencies, such as data literacy, algorithmic thinking, ethical considerations, and critical evaluation of AI‐generated outputs [27, 35]. This trend has been reflected in survey‐based studies and competency‐mapping efforts that underscore growing institutional efforts to embed these themes into both undergraduate and postgraduate training programs [25, 28]. Training modules increasingly included exposure to tools such as predictive analytics, machine learning techniques, and natural language processing, particularly in the context of disease surveillance, health communication, and decision support systems [32]. These initiatives aimed to demystify complex AI concepts and integrate them into foundational public health education [26, 34].

Disparities in digital competency were particularly evident among professionals engaging in mid‐career or continuing education programs [30]. In such instances, customized instructional strategies were required to address diverse learner needs and backgrounds [28, 37]. Early introduction of AI concepts in undergraduate curricula was recognized as a promising approach to ensure baseline digital proficiency and to prepare students for more advanced learning and application later in their careers [21, 38].

### 3.4 Institutional Readiness and Technological Infrastructure

The capacity of educational institutions to adopt AI‐based tools varied considerably, highlighting the importance of institutional readiness and digital infrastructure. Institutions with well‐developed information and communication technology (ICT) systems were better positioned to implement AI‐enhanced teaching methods [24, 43]. They often demonstrated strategic planning, investment in digital platforms, and supportive policies for technological integration [26]. Conversely, institutions operating in resource‐constrained environments faced significant obstacles, including unreliable internet connectivity, outdated hardware, and limited budgets [28]. The lack of dedicated funding for AI integration further exacerbated these challenges [32, 35]. To overcome these barriers, partnerships with private sector entities and government bodies were frequently recommended to support infrastructure development and ensure equitable access to AI technologies [26].

In addition to infrastructure, institutional policies played a key role in guiding the adoption of AI. Governance frameworks addressing responsible and ethical AI use were instrumental in fostering trust and acceptance [21, 44]. These policies often included provisions for faculty training, student data protection, and ethical oversight of AI‐driven educational research [23]. Innovative tools such as learning analytics dashboards were also introduced to monitor student performance, personalize feedback, and support early intervention for at‐risk learners—though concerns about privacy and autonomy remained areas of active debate [29, 36].

### 3.5 Ethical Considerations and Equity in AI Integration

Ethical reflection emerged as a central concern in the integration of AI into public health education. Given the field's foundational commitment to social justice and health equity, the deployment of AI tools in academic settings necessitated careful examination of their broader implications [32]. Key ethical issues included algorithmic bias, data security, informed consent, and unequal access to digital resources [24]. If left unaddressed, these factors had the potential to exacerbate existing inequalities, particularly for students from marginalized communities [25]. Limited internet access, linguistic barriers, and socioeconomic constraints created additional obstacles for students in underrepresented or rural areas, raising serious concerns about inclusivity and fairness [29].

Institutions varied in their approaches to addressing these ethical challenges [39]. Some integrated AI ethics into their curricula, equipping students with the tools to critically assess and responsibly use emerging technologies [31, 46]. However, these efforts were inconsistent and typically concentrated within institutions with pre‐existing strengths in digital ethics or technology studies [42]. There was a growing consensus on the need for standardized ethical frameworks co‐developed by educators, technologists, and ethicists [22, 41]. Such frameworks were envisioned to ensure transparency, accountability, and equity in AI adoption while promoting informed participation and ethical awareness among students and faculty alike [30, 38].

### 3.6 Interdisciplinary Collaboration and Curriculum Innovation

AI's introduction into public health education spurred widespread interest in interdisciplinary collaboration and curriculum reform. Many academic programs began fostering partnerships between public health departments and disciplines, such as computer science, data analytics, behavioral science, and health informatics [25]. These cross‐disciplinary initiatives enabled the co‐creation of AI‐integrated courses that reflect the complexity of modern public health challenges [39]. Curricular innovations included embedding AI‐related modules into existing courses on epidemiology, global health, and health systems management [33]. These updates promoted active learning through collaborative projects, problem‐solving exercises, and experiential learning opportunities [40]. Co‐teaching arrangements and project‐based learning models were adopted to bring together expertise from different fields, enriching the educational experience [41].

Furthermore, collaboration with industry stakeholders and public health organizations became increasingly valuable in aligning educational content with workforce needs [31]. These partnerships supported the development of real‐world training environments, internships, and applied research projects, facilitating practical exposure to AI tools in public health settings [42, 45]. Despite these advances, interdisciplinary efforts were sometimes hindered by structural barriers such as departmental silos and disciplinary hierarchies [25, 39]. Some faculty members expressed concerns about the potential overemphasis on technological solutions at the expense of human‐centered approaches [22]. These tensions underscored the need for inclusive, balanced curriculum development processes that honor the values of both technological innovation and public health equity [40].

## 4 Discussion

This scoping review catalogs the current body of literature describing how artificial intelligence (AI) has become an influential driver in the evolving landscape of public health education. Rather than a wholesale system‐wide transformation, the literature reflects a gradual but notable shift from traditional teaching methods to AI‐enhanced pedagogical models. Tools such as intelligent tutoring systems, adaptive learning platforms, and virtual simulations have made it possible to personalize instruction, increase engagement, and simulate real‐world public health scenarios. These findings are consistent with those of Farhud and Zokaei, who examined ethical challenges related to AI in medicine and healthcare, rather than directly evaluating educational outcomes [47]. While their work does not measure learning impact, it contributes meaningfully to ongoing discussions on the ethical frameworks necessary for responsible AI adoption in education. AI‐driven simulations allowed learners to interact with complex public health emergencies in a risk‐free environment, fostering skills such as epidemiological modeling and critical thinking [48]. In contrast, Mellado et al. (2021) explored how AI and big data were used to inform COVID‐19 public health strategies in Africa but did not focus on comparing immersive learning with passive instructional methods [49].

Equally critical is the role of AI in building digital and analytical competencies among both students and educators. As identified in this review, educational institutions have increasingly recognized the importance of integrating data literacy, algorithmic thinking, and ethical reasoning into public health curricula. This aligns with the work of Musbahi et al. (2021), who argued that future public health professionals should be equipped to interpret and critique AI outputs [50]. Training that introduces concepts like machine learning, predictive analytics, and natural language processing was frequently described as foundational in reviewed studies, particularly for applications in health communication and disease surveillance [51]. Webster and Neal (2024) reported that the inclusion of AI modules led to improved learner performance in courses involving health data analysis [52]. However, the review also describes observable gaps in digital preparedness, especially among mid‐career professionals who often lacked foundational skills [53]. Tailored instructional strategies and continuous education pathways are therefore crucial to ensure that all learners, regardless of background, can adapt to AI‐enhanced environments [54].

The review further underscores the significance of institutional readiness in the successful implementation of AI in public health education. Institutions with robust digital infrastructure, strategic planning, and clear policies for technology integration appeared to demonstrate greater success in adopting AI tools, as interpreted from descriptive and implementation‐focused studies included in this review. Ayenigbara (2024) noted similar findings, emphasizing that institutional capacity, including technical support, training, and governance, was essential to embedding AI sustainably into educational frameworks [55]. In contrast, organizations with limited resources faced challenges such as poor connectivity, outdated equipment, and a lack of dedicated funding for technological innovation [56]. These disparities were noted as a potential contributor to inequitable educational access and prompted calls in the literature for collaborative strategies [57]. Recommendations in the literature suggest fostering partnerships between educational entities, technology providers, and public institutions to support equitable access and infrastructure development [58]. The availability of learning analytics dashboards and intelligent feedback tools has shown promise in personalizing support for students, though concerns about data privacy and autonomy remain significant [59].

Ethical considerations emerged as a central theme in this review, particularly given public health's inherent focus on equity and social responsibility. The integration of AI into education has introduced complex ethical questions, including algorithmic bias, informed consent, and fair access to digital resources. Dankwa‐Mullan (2024) raised alarms about the risk of biased algorithms perpetuating systemic inequities, an issue that holds similar relevance in educational settings where digital tools may unintentionally disadvantage underrepresented learners [60]. Cresswell et al. (2021) emphasized that ethics education should be embedded throughout AI‐focused curricula to cultivate responsible digital citizenship [61]. Although some institutions have begun implementing such measures, the literature indicated inconsistencies in approach, limiting their broader applicability [62]. The findings of this review support calls for a standardized, cross‐disciplinary ethical framework to guide AI use in education. Such frameworks would not only mitigate potential harm but also empower students and educators to engage with AI critically and confidently.

Furthermore, the review highlights a growing trend of interdisciplinary collaboration and curriculum innovation prompted by AI integration. By fostering collaboration between departments such as public health, computer science, behavioral science, and data analytics, institutions have begun creating hybrid learning environments that reflect the complexity of real‐world health challenges. Dong et al. (2021) found that interdisciplinary teaching models, particularly those involving project‐based and experiential learning, enhanced students' understanding of AI's applications in public health [63]. However, structural challenges such as disciplinary silos and faculty resistance remain obstacles to sustained reform [64]. Some educators, as reported in the reviewed studies, expressed concern that technological emphasis could shift attention away from the human dimensions of public health practice [65]. Therefore, curriculum development should strike a balance by honoring technological progress while also acknowledging the foundational values of empathy, community engagement, and health equity [66]. Strengthening collaborations with public health agencies and industry partners has also proven effective in aligning academic instruction with workforce needs, preparing students for practical, AI‐enhanced roles in the evolving public health landscape [67].

### 4.1 Policy and Regulatory Implications

The integration of artificial intelligence (AI) into public health education necessitates comprehensive policy and regulatory responses that align with evolving educational and technological landscapes. As AI tools become embedded in curricula, policymakers should establish clear, consistent guidelines to ensure their responsible use. These regulations should go beyond technical specifications and address broader concerns such as algorithmic fairness, data protection, transparency, and ethical oversight, as emphasized in studies [13, 15, 60]. In many regions, especially those with limited digital infrastructure, policy gaps can exacerbate inequities, creating an uneven playing field for both institutions and learners [29, 35]. Therefore, equitable access to AI resources should be a cornerstone of regulatory planning. Studies also highlight the need for participatory governance mechanisms and adaptive policies that respond to the evolving AI landscape in education [12, 44]. National and institutional policies should include strategies for faculty training, institutional capacity‐building, and continuous monitoring of AI's impact on educational quality and inclusion. Governance mechanisms should promote accountability and participatory decision‐making, incorporating diverse voices from educators, technologists, ethicists, and students. Accreditation bodies and academic councils are also pivotal in ensuring the standardization of AI competencies across curricula [24]. Additionally, flexible regulatory frameworks are needed to adapt to the rapid evolution of AI tools, without stifling innovation. Investment in public–private partnerships can help bridge infrastructure gaps and foster collaborative innovation. Ultimately, well‐defined policies and ethical safeguards are essential not only to guide responsible AI adoption but also to ensure that these technologies advance public health education in a manner that is fair, inclusive, and socially responsible.

### 4.2 Recommendations for Curriculum Reform

Curriculum reform is essential to ensure that public health education evolves in tandem with the rapid advancements in artificial intelligence. Institutions should adopt a holistic and interdisciplinary approach, embedding AI‐related competencies throughout the educational continuum. As indicated by the mapped studies [25, 27, 28], this includes introducing foundational digital literacy and algorithmic thinking at the undergraduate level, followed by advanced modules on machine learning, natural language processing, and ethical AI application in postgraduate and continuing education programs. Public health curricula should be redesigned to include hands‐on experience with AI tools used in real‐world settings, such as predictive analytics for disease surveillance or decision support systems in policy modeling. This was a recommendation found across several implementation‐focused studies [30, 34, 38].

Collaboration between departments, particularly public health, computer science, behavioral science, and ethics, was emphasized in both the included studies [31, 39, 42] and supported by established frameworks such as those proposed by Russell et al. (2023), suggesting that institutionalized interdisciplinary efforts create more integrated and future‐relevant learning experiences [28]. Problem‐based learning, project‐based assignments, and AI‐powered simulations should be leveraged to promote critical thinking and practical engagement. This pedagogical approach was identified in both expert perspectives and descriptive studies [23, 27, 39]. Additionally, educators need support through structured faculty development programs that build their confidence and capacity to teach AI‐related content effectively. Addressing gaps in digital preparedness, especially among mid‐career professionals through tailored instructional strategies, was a need highlighted by both survey‐based studies and expert interpretation [28, 32, 36, 37]. Ultimately, drawing from both empirical literature and conceptual recommendations, reforming curricula in this way will not only produce technologically competent graduates but also cultivate a workforce capable of navigating the ethical, social, and operational complexities of AI in public health practice.

### 4.3 Future Research Directions

Future research should delve deeper into understanding the long‐term impacts of artificial intelligence on learning outcomes, workforce readiness, and ethical development within public health education. While current literature highlights promising applications of AI in pedagogy, empirical evidence on its sustained effectiveness remains limited. Longitudinal studies are needed to assess how AI‐enhanced education influences professional performance, decision‐making capabilities, and equity in career progression. Additionally, research should investigate the contextual factors that affect AI adoption, particularly in low‐resource educational environments where technological infrastructure may be lacking. Comparative studies across geographic, cultural, and institutional contexts will help identify scalable and culturally sensitive implementation models. Another priority area involves exploring the intersection of AI ethics and education, specifically how students internalize ethical reasoning when exposed to automated decision‐making systems. Research should also examine the role of interdisciplinary partnerships in shaping curriculum and fostering innovation. Evaluating faculty experiences and barriers to teaching AI‐related content will offer insights for designing effective professional development programs. Moreover, participatory research that includes students' voices can illuminate the nuanced challenges they face and inform the co‐creation of inclusive learning environments. Advancing these research agendas will be crucial to building a resilient, ethically grounded, and digitally fluent public health workforce.

## 5 Conclusion

This scoping review highlights the transformative role of artificial intelligence in reshaping public health education. AI technologies, ranging from virtual simulations to adaptive learning platforms, have enhanced the delivery of education, enabling more personalized, interactive, and competency‐driven learning experiences. The integration of AI has supported the development of critical skills such as epidemiological modeling, digital literacy, and ethical decision‐making, positioning future public health professionals to navigate increasingly complex health landscapes. Drawing upon the reviewed literature, key findings reveal that AI‐facilitated pedagogical innovations were associated with improved learner engagement and practical skill development, particularly in areas such as real‐time public health decision‐making and disease surveillance. The emergence of AI‐specific competency development was evident through curricular emphasis on data literacy, algorithmic reasoning, and ethical awareness. However, institutional disparities in digital infrastructure and faculty capacity were consistently reported as barriers to equitable implementation.

Despite these promising trends, notable evidence gaps remain. These include the absence of longitudinal evaluations on AI's sustained impact on learning outcomes and a lack of research from low‐resource educational settings. Ethical concerns, including algorithmic bias, data privacy, and inequitable access, were frequently mentioned but not systematically addressed across studies. This underscores the need for stronger governance frameworks and standardized ethical curricula. Therefore, conclusions drawn from the current body of literature emphasize the necessity of interdisciplinary collaboration, participatory policy development, and faculty capacity‐building to ensure sustainable and inclusive AI integration. As AI continues to evolve, its thoughtful and equitable integration into public health education will be essential for building a digitally fluent, ethically grounded, and practice‐ready workforce. Future initiatives should prioritize empirical evaluation, inclusive design, and global equity to ensure AI meaningfully contributes to the goals of public health.

## Author Contributions

Conceptualization: Mst Masuma Akter Semi, Srabani Das, Mashuk Rahman Utsho, Sumaiya Yeasmin, and Mst. Rina Parvin. Methodology: Mst Masuma Akter Semi, Srabani Das, Mashuk Rahman Utsho, Adib Hossain, Arif Ahmed Sizan, and Mst. Rina Parvin. Software: Mst Masuma Akter Semi, Sumaiya Yeasmin, and Mst. Rina Parvin. Data curation: Mst Masuma Akter Semi, Srabani Das, Mashuk Rahman Utsho, Adib Hossain, Md Bayzid Kamal, Afia Fairooz Tasnim, Sumaiya Yeasmin, and Mst. Rina Parvin. Investigation: Mashuk Rahman Utsho, Arif Ahmed Sizan, Afia Fairooz Tasnim, and Sumaiya Yeasmin. Validation: Mashuk Rahman Utsho and Afia Fairooz Tasnim. Formal analysis: Mst Masuma Akter Semi, Mashuk Rahman Utsho, Adib Hossain, Md Bayzid Kamal, Arif Ahmed Sizan, Afia Fairooz Tasnim, and Mst. Rina Parvin. Supervision: Srabani Das, Sumaiya Yeasmin, and Mst. Rina Parvin. Visualization: Srabani Das, Adib Hossain, and Sumaiya Yeasmin. Project administration: Mst Masuma Akter Semi, Srabani Das, Adib Hossain, Md Bayzid Kamal, Afia Fairooz Tasnim, and Mst. Rina Parvin. Resources: Mst Masuma Akter Semi, Srabani Das, Adib Hossain, Arif Ahmed Sizan, Afia Fairooz Tasnim, Sumaiya Yeasmin, and Mst. Rina Parvin. Writing – original draft: Mst Masuma Akter Semi, Mashuk Rahman Utsho, Md Bayzid Kamal, and Mst. Rina Parvin. Writing – review and editing: Mst Masuma Akter Semi, Srabani Das, Adib Hossain, Md Bayzid Kamal, Arif Ahmed Sizan, Afia Fairooz Tasnim, and Mst. Rina Parvin. All authors have read and approved the final version of the manuscript.

## Funding

The authors received no specific funding for this work.

## Disclosure

The lead author Mst. Rina Parvin affirms that this manuscript is an honest, accurate, and transparent account of the study being reported; that no important aspects of the study have been omitted; and that any discrepancies from the study as planned (and, if relevant, registered) have been explained.

## Ethics Statement

The authors have nothing to report.

## Consent

Informed consent was obtained from all individual participants surveyed in the study.

## Conflicts of Interest

The authors declare no conflicts of interest.

## Data Availability

Data sharing is not applicable to this article, as no new data were created or analyzed in this study.
